# One-Year Handgrip Strength Change in Kindergarteners Depends upon Physical Activity Status

**DOI:** 10.3390/life13081665

**Published:** 2023-07-31

**Authors:** Akemi Abe, Rika Sanui, Jeremy P. Loenneke, Takashi Abe

**Affiliations:** 1 Division of Children’s Health and Exercise Research, Institute of Trainology, Fukuoka 814-0001, Japan; amyabe3379@gmail.com; 2 Child Health Research Group, Atagohama Kindergarten, Fukuoka 819-0002, Japan; atagohama1@gmail.com; 3 Department of Health, Exercise Science, & Recreation Management, Kevser Ermin Applied Physiology Laboratory, The University of Mississippi, Oxford, MS 38677, USA; jploenne@olemiss.edu; 4 Institute of Health and Sports Science & Medicine, Graduate School of Health and Sports Science, Juntendo University, Inzai 270-1695, Japan

**Keywords:** grip strength, young children, active play, growth and development

## Abstract

Free play in kindergarten can be roughly divided into fine and gross motor activities, but the effects of these activities on improving handgrip strength are unknown. Therefore, we aimed to compare one-year changes in handgrip strength and forearm flexor muscle size in children separated by preferred play in a kindergarten. One hundred and eleven children were recruited from a local kindergarten. They underwent handgrip strength and forearm muscle thickness measurements, and 95 (49 boys and 46 girls) underwent a second measurement one year after the first measurement. Class teachers assessed the physical activity of everyone in their class after the second measurement. Using three evaluation scores by the class teachers, we divided children into three groups based on the children’s preference to play in kindergarten (fine movement vs. gross motor movement). Handgrip strength did not change differently between groups across one year. However, children who liked active playing outside (i.e., gross motor activity) were stronger than others. Furthermore, children who like playing outside observed greater changes than the other groups in the ulna (right hand) and radius muscle thickness (left hand), suggesting that changes in forearm muscle size might be incongruent with changes in handgrip strength among the three activity groups.

## 1. Introduction

Handgrip strength is a biomarker of current health status and future morbidity/disability in middle-aged and older adults [1]. For example, a study investigated the association between handgrip strength and the incidence of heart disease in 20,829 middle-aged and older adults aged 50 or older [2]. The incidence rate of heart disease was adjusted for covariates such as age, education level, physical activity, body mass index, alcohol, smoking, and medical history. The authors found that those in the highest quartile of handgrip strength had a 35% lower risk of being diagnosed with heart disease compared with those in the lowest quartile in men and 46% in women over 13 years of follow-up. In addition, it is noted that there was a difference of 12 kg for men and 10 kg for women between the highest and lowest quartile cut-off values for handgrip strength in each age group. Similarly, recent large-sized follow-up studies also reported an inverse association between handgrip strength and the incidence rate of type 2 diabetes [3], cancer [4], and dementia [5]. Our article search found over 100 similar articles in the last quarter century (e.g., [6,7,8,9,10,11,12,13,14,15,16,17,18,19,20,21,22,23,24,25]).

Given that handgrip strength can be a biomarker that predicts current and future health status, it seems necessary to discuss how to increase this handgrip strength [26,27]. Recently, we compared differences in handgrip strength among different sports in first-year university male students enrolled in a sports university [28]. We found that handgrip strength was greater in participants who participated in sports with upper-body movements (i.e., kendo and baseball) than those in sports that primarily involve lower-body activities (i.e., soccer). Interestingly, the mean difference between the kendo and soccer groups was approximately 5 kg, but the difference between the two groups has gradually widened over the past 45 years. Similar results were observed in female students [29]. Those results suggest that the type of physical activity performed during the developmental period may affect the handgrip strength acquired by children in the development process.

Although research is limited, previous studies have examined the impact of family- and school-based interventions on handgrip strength in children and adolescents [30,31,32]. However, the intervention program did not affect handgrip strength in the intervention group compared with the control group. Unfortunately, most studies investigating the effects of school-based physical education classes did not include handgrip strength changes after the exercise intervention [27]. In addition, more recent studies investigating the effects of an exercise training intervention on handgrip strength in preschool-aged children have reported conflicting results; one study found significant improvements in handgrip strength in the intervention group compared to the control group [33], while another did not [34]. However, we could not determine why one study found a benefit and another did not.

In free-play behaviors in kindergarten, some children prefer fine movements (primarily using hands and fingers) and others prefer gross motor activities (primarily using the lower body). It would be meaningful to know the impact of preferred play on changes in handgrip strength during growth. Therefore, this study aimed to compare one-year changes in handgrip strength and forearm muscle size in children separated by preferred play in a kindergarten (fine movement vs. gross motor movement).

## 2. Materials and Methods

### 2.1. Study Design

This is a study of kindergarteners conducted in the city of Fukuoka, Japan. This kindergarten has a relatively large (approximately 3100 m^2^) lawn garden for children. Data collection took place from October 2021 to November 2022. The measurements were performed in the morning (9:00–10:00 AM, at room temperature of approximately 22 °C) using the same methodological protocols described below. This study received approval from the ethics committee of Seinan Gakuin University (application no SG #2021-2-2) and was conducted according to the Declaration for Helsinki. Children with their parents were fully informed about the purpose of the study and its safety, and written informed consent was obtained from the parents of each child.

### 2.2. Participants

With the cooperation of the school’s staff and parents, 111 young children (56 boys and 55 girls) were recruited from a local kindergarten. Few children used their left hand or mixed hands to eat and write (*n* = 4). All participants completed the first measurement, but 95 (49 boys and 46 girls) underwent a second measurement one year after the first because some children transferred to other kindergartens due to their parents’ jobs (Table 1). Only data from these 95 participants were used for this study.

### 2.3. Handgrip Strength Measurements

Maximum voluntary handgrip strength was measured with the right and left hands using a Smedley handgrip dynamometer (TKK Grip-A, Niigata, Japan) [35,36]. All children were instructed to maintain an upright standing position to keep their arms at their sides. The participants held the dynamometer in their right or left hand with the elbow extended downward without squeezing. The distance of the dynamometer grip bars (grip span) was adjusted to the hand size of the children (the middle phalanx rested on the inner handle) [37]. All children were allowed to perform two maximal trials on each side with a one-minute break. All the participants appeared motivated during the strength tests [38,39]. The highest value on each side was used for data analysis.

### 2.4. Forearm Muscle Thickness Measurements

Anterior forearm muscle thickness was also measured using B-mode ultrasound (Logiq e; GE, Fairfield, CT, USA) at 30% proximal of forearm length (between the styloid process and the head of the radius) on the right side of the body [40]. A linear scanning head was coated with transmission gel and placed on the skin surface of the measurement site with minimum pressure to achieve a clear image. Two images from the site were stored for offline analysis following data collection. Muscle thickness of the radius (MT-radius) and ulna (MT-ulna) was measured as the perpendicular distance between the adipose tissue–muscle interface and the muscle–bone interface. The average value measured on two images was used for data analysis.

Before ultrasound measurements, standing height and body mass were measured to the nearest 0.1 cm and 0.1 kg, respectively, using a height scale and an electronic weight scale. Forearm length and girth (at 30% proximal of forearm length) were also measured using a flexible tape measure.

### 2.5. Physical Activity Assessments

Class teachers assessed the physical activity of everyone in their class after the second measurement. The teacher of each class was unaware of any of the study participants’ results from the handgrip strength test and ultrasound muscle thickness measurements. We asked the class teachers to evaluate the physical activity level from the following three evaluation points. The first evaluation point concerned whether the children preferred to play indoors or outdoors during free play in kindergarten: (1) Often plays indoors, (2) intermediate, and (3) often plays outdoors. The second evaluation point was whether or not children sweated during free play: (1) Never sweating from play, (2) intermediate, and (3) always sweating from play. The last evaluation point was a question about the type of play. We provided teachers with two different examples of play: One being gross-motor movements (e.g., tag, rope-jumping, dodgeball) and the other being fine movements (e.g., block play, the art of paper folding, drawing pictures). We asked teachers whether or not children were challenged with various play in each preferred play: (1) Often performed the same type of play; (2) intermediate; (3) or performed many different types of challenging play.

From the above three points, we divided them into three groups. For example, children who preferred to sit and play, never sweated from play, and performed relatively similar activities would be rated 1, 1, and 1 and would be assigned to “Group 1”. In contrast, children who liked to play outdoors, always sweated from play, and engaged in many types of play would be rated 3, 3, and 3 and would be assigned to “Group 3”. Children rated 2 (intermediate) on three evaluation points were assigned to “Group 2.” When all three scores were not the same, children were grouped as follows: No children had all different ratings (i.e., 1, 2, and 3) on the three evaluation points. That is, at least two ratings were in agreement. Therefore, we adopted two matching evaluation points to separate the groups. As a result, the number of children in each group was divided as follows: Group 1 (*n* = 23), Group 2 (*n* = 34), and Group 3 (*n* = 38).

### 2.6. Statistical Analysis

Overall changes in handgrip strength and muscle size were determined using paired sample *t*-tests. A repeated-measures ANOVA on time with a between-subject factor of sex was used to determine differences in muscle size and strength between boys and girls. To determine the influence of activity level, we used a repeated-measures ANOVA on time with a between-subject factor of activity level. We also included sex as a covariate to determine if that altered the result. Post-hoc comparisons were not adjusted for multiple comparisons. Statistical significance was set at *p* < 0.05. Normality was visually assessed using a Q-Q plot, and Levene’s test was used for testing the homogeneity of variance. All statistical analyses were run using Jamovi version 2.3.13.0. Data are presented as mean and 95% confidence intervals unless otherwise stated.

## 3. Results

### 3.1. Overall Change in Handgrip Strength and Muscle Size

Handgrip strength increased in both the right (a change of 3.3 (3.0, 3.7) kg) and left (a change of 3.1 (2.7, 3.4) kg) hands. For muscle thickness, there were also increases for MT-ulna (right forearm: 1.9 (1.7, 2.1) mm; left forearm: 1.8 (1.6, 2.1) mm) and MT-radius (right forearm: 0.7 (0.5, 0.8) mm; left forearm: 0.7 (0.5, 0.9) mm) in both the right and left forearms.

### 3.2. Comparison between Boys and Girls

Handgrip strength did not change differently between boys and girls for either the right hand (time x sex: *p* = 0.498) or the left hand (time x sex: *p* = 0.991). Handgrip strength was greater at the second time point compared to the first. However, boys had greater overall strength than girls (when collapsed across time; *p* ≤ 0.001). On the right hand, boys were 1.6 (0.7, 2.5) kg stronger, and on the left hand, boys were 1.5 (0.6, 2.4) kg stronger (Table 1).

MT-ulna did not change differently between boys and girls (Table 1) for either the right forearm (time x sex: *p* = 0.309) or the left forearm (time x sex: *p* = 0.388). Muscle thickness was greater at the second time point than the first, but there was no main effect of sex (*p* = 0.11 and *p* = 0.1 for right and left, respectively).

MT-radius did not change differently between boys and girls (Table 1) for either the right forearm (time x sex: *p* = 0.91) or the left forearm (time x sex: *p* = 0.388). Muscle thickness was greater at the second time point than the first, but there was no main effect of sex (*p* = 0.06 and *p* = 0.1 for right and left, respectively).

### 3.3. Comparison among Three Physical Activity Groups

**Figure 1:**
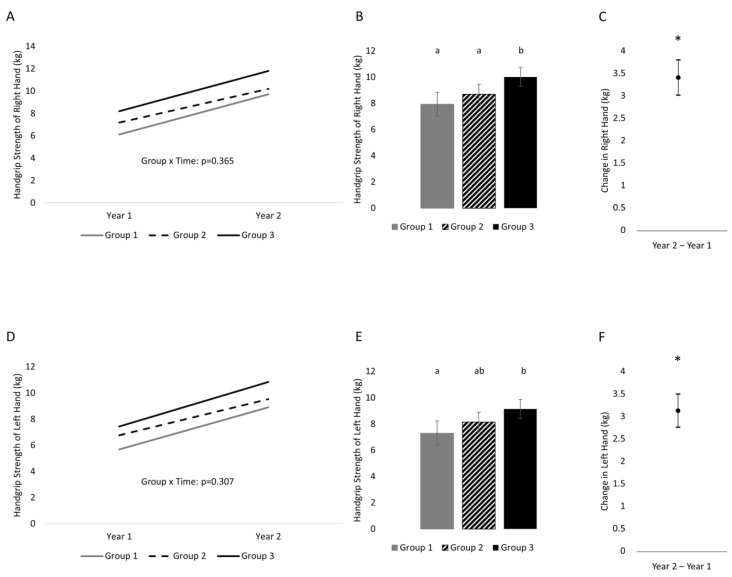
One-year changes in handgrip strength of the right and left hands separated into three groups on the basis of physical activity status. (**A**) Changes across time separated by group (variability of changes are found in Table 2) in the right hand, (**B**) between-subject effect of group (collapsed across time) in the right hand, (**C**) time effect (collapsed across group) in the right hand; (**D**) change across time separated by group (variability of changes are found in Table 2) in the left hand, (**E**) between-subject effect of group (collapsed across time) in the left hand, (**F**) time effect (collapsed across group) in the left hand. Variability is represented by 95% confidence intervals. Conditions that share a letter are not statistically significantly from each other. * indicates a statistically significant time effect.

Handgrip strength did not change differently between activity levels for either the right hand (time x activity level: *p* = 0.365) or the left hand (time x activity level: *p* = 0.307) (Figure 1). Handgrip strength was greater at the second time point compared to the first; however, activity level did influence overall strength when collapsed across time for the right hand (*p* = 0.002) and the left hand (*p* = 0.008). Post-hoc analysis for the right hand found that Group 3 was significantly greater than Group 2 (difference of 1.3 (0.2, 2.3) kg, *p* =0.015) and Group 1 (difference of 2 (0.9, 3.2) kg, *p* = 0.001). Group 1 was not statistically different from Group 2 (*p* = 0.2). This result held following an adjustment for sex. Post-hoc analysis for the left hand found that Group 3 was significantly greater than Group 1 (difference of 1.8 (0.6, 3.0) kg, *p* = 0.002) but not Group 2 (difference of 0.9 (−0.05, 2.0) kg, *p* = 0.06). Group 2 was also not statistically different from Group 1 (*p* = 0.166). This result held following an adjustment for sex.

**Figure 2:**
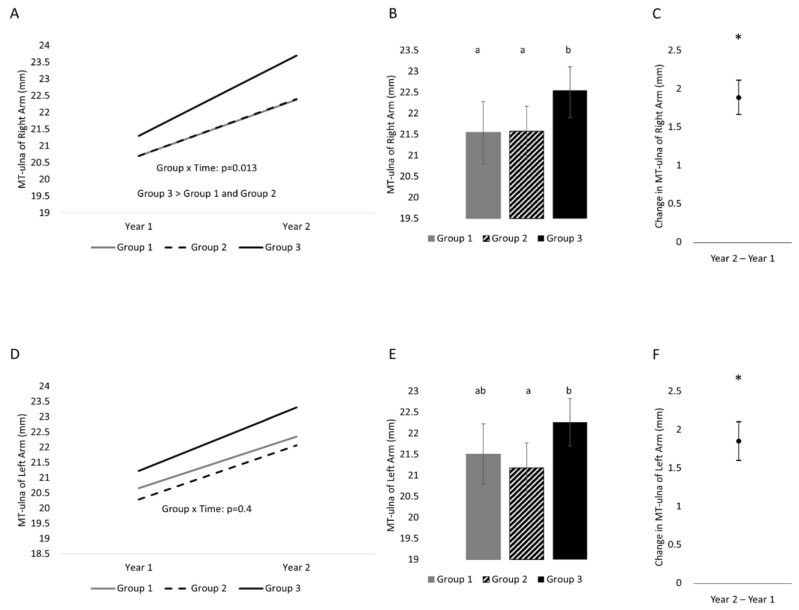
One-year changes in MT-ulna of the right and left hands separated into three groups on the basis of physical activity status. (**A**) Changes across time separated by group (variability of changes are found in Table 2) in the right arm, (**B**) between-subject effect of group (collapsed across time) in the right arm, (**C**) time effect (collapsed across group) in the right arm; (**D**) change across time separated by group (variability of changes are found in Table 2) in the left arm, (**E**) between-subject effect of group (collapsed across time) in the left arm, (**F**) time effect (collapsed across group) in the left arm. Variability is represented by 95% confidence intervals. Conditions that share a letter are not statistically significantly from each other. * indicates a statistically significant time effect.

MT-ulna changed differently (Figure 2) between activity levels for the right forearm (time x activity level: *p* = 0.013) but not the left forearm (time x activity level: *p* = 0.4). Changes across the year were greatest in Group 3 compared to Group 2 (difference of 0.67 (0.17, 1.1) mm) and Group 1 (difference of 0.68 (0.11, 1.2) mm). There were no differences between Group 2 and Group 1 (*p* = 0.986). The statistical interaction held following an adjustment for sex. When collapsed across time, Group 3 was greater than Group 2 (*p* = 0.02) and Group 1 (*p* = 0.03), but Group 2 was not greater than Group 1 (*p* = 0.96). For MT-ulna of the left forearm, there was a time effect and effect of activity level. MT-ulna was greater at the second time point than the first, and Group 3 was greater than Group 2 (difference of 1.0 (0.26, 1.9) mm, *p* = 0.01). There were no other statistically significant differences. Results remained the same following an adjustment for sex.

**Figure 3:**
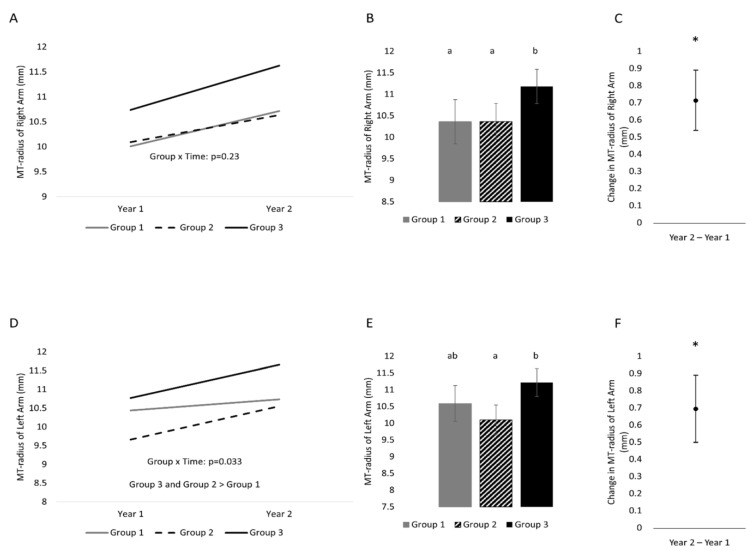
One-year changes in MT-radius of the right and left hands separated into three groups on the basis of physical activity status. (**A**) Changes across time separated by group (variability of changes are found in Table 2) in the right arm, (**B**) between-subject effect of group (collapsed across time) in the right arm, (**C**) time effect (collapsed across group) in the right arm; (**D**) change across time separated by group (variability of changes are found in Table 2) in the left arm, (**E**) between-subject effect of group (collapsed across time) in the left arm, (**F**) time effect (collapsed across group) in the left arm. Variability is represented by 95% confidence intervals. Conditions that share a letter are not statistically significantly from each other. * indicates a statistically significant time effect.

MT-radius did not change differently (Figure 3) between activity levels for the right forearm (time x activity level: *p* = 0.23), but it did for the left forearm (time x activity level: *p* = 0.033). Both results held following an adjustment for sex. MT-radius of the right forearm was greater at the second time point than the first. Group 3 had greater MT-radius values than Group 2 (difference of 0.81 (0.23, 1.39) mm; *p* = 0.006) and Group 1 (difference of 0.82 (0.16, 1.47) mm; *p* = 0.014). Group 2 was not different from Group 1 (*p* = 0.993). MT-radius of the left arm changed differently across time between activity levels. Changes across the year in the left forearm were greater in Group 3 and Group 2 compared to Group 1 (Group 3 vs. Group 1: 0.59 (0.1, 1.0) mm; Group 2 vs. Group 1: 0.59 (0.09, 1.0) mm). There was no difference in the differences between Group 3 and Group 2. When collapsed across time, Group 3 was greater than Group 2 (*p* = 0.0004).

## 4. Discussion

We investigated whether the change in one-year handgrip strength depended upon the children’s physical activity status. Physical activity status was based on the children’s preferred play in kindergarten (fine movement vs. gross motor movement). The present study found that a one-year change in handgrip strength was similar among three physical activity groups. However, children who liked active play outside (i.e., gross motor movement) were stronger than children of other groups (i.e., group effect). In contrast, muscle thickness changed differently across groups in MT-ulna of the right hand and MT-radius of the left hand. These results suggest that differences among children already exist as early as kindergarten and that changes in forearm muscle thickness do not always occur in alignment with changes in muscle strength.

### 4.1. One-Year Change in Handgrip Strength

Active play that children perform outside in kindergarten during free time is an activity that primarily uses the lower body. Previous studies [41,42] observed increased muscle strength of the untrained upper extremities due to resistance training of the lower extremities (called the cross-transfer effect). However, there are no studies on increased handgrip strength with a lower-body exercise intervention, and no such results were found in this study. On the other hand, fine motor activities primarily use the hands and fingers. Therefore, practicing fine motor activities helps children improve their fine motor skills [43].

In the present study, our results showed that a one-year change in handgrip strength was not different among the three active groups, but children who liked active play outside (Group 3) were stronger than other groups. Therefore, children in Group 3 are hypothesized to have been similarly active before the study period, which may have contributed to their greater handgrip strength. Unfortunately, in this study, we could not evaluate the specific play of children, such as types, amount, and intensity before and during this study. Furthermore, we could not investigate what children do with their families after school or on weekends. However, previous studies have reported the possibility that exercise using the upper body during play in children may be involved in improving handgrip strength [27]. For example, one study investigated the effects of upper-body exercise on handgrip strength in an intervention group by comparing it with a control group [44]. The intervention group (*n* = 40, mean age 8.4 years) performed upper-body exercises such as self-supported movements (e.g., wheelbarrow, seal walk, crabwalk) and circuit exercises (e.g., tennis balls for squeezing, strips of rubber tire to pull) three times a week. The control group (*n* = 46, mean age 8.6 years) had a free-play period, which was part of the normal school routine. The changes in handgrip strength were significantly greater (*p* < 0.05) for the intervention group compared to the control group (pre–post change of the right hand: Experimental group = 1.5 kg and control group = 0.3 kg) following a 12-week intervention. More recently, we investigated the effects of the type of sports practiced on handgrip strength in first-year sport university students, as mentioned above [28,29]. The authors selected two types of sporting events with matching physiques (i.e., height and body mass); Soccer (*n* = 1127) targets the lower body, and Kendo (*n* = 297) and Baseball (*n* = 698) use the lower body simultaneously with upper-body movement (including gripping) and report that those in the lower-body-only (Soccer) sports had −3.78 (95% CI: −4.27, −3.29) kg lower handgrip strength than those in the lower + upper body (Kendo and Baseball) sporting events [28]. Comparing each individual sport found that each sport was different from the others with Kendo > Baseball > Soccer (between each sport, *p* < 0.001). In addition, the difference in handgrip strength between Kendo and Soccer was approximately 5 kg in the overall sample. Considering the results of the above-mentioned previous studies and this study, it is expected that grip strength during an object’s gripping movements improves handgrip strength during the developmental period. However, further research is needed on the gripping conditions (e.g., intensity and duration) for improving handgrip strength in children and adolescents.

### 4.2. One-Year Change in Forearm Muscle Thickness

An interesting finding obtained in this study was that changes in handgrip strength and changes in forearm flexor muscle thickness were inconsistent among the three groups with different physical activity preferences. We recently investigated the association between handgrip strength changes and forearm flexor muscle thickness changes in 218 young children different from the sample of this study [45]. The study found that there were significant (*p* < 0.001) within-subject correlations between ulna muscle thickness and handgrip strength (r = 0.50) and radius muscle thickness and handgrip strength (r = 0.59). However, while there was a statistically significant (*p* < 0.001) between-subject relationship between radius muscle thickness and handgrip strength (r = 0.27), there was no significant between-subject correlation between ulna muscle thickness and handgrip strength (r = 0.07). It has been reported that muscle strength and size changes do not always match during resistance training in healthy young adults [46]. Although changes do not necessarily line up, it should be noted that those in Group 3 tended to have the most muscle size and strength.

The reasons for the difference in muscle thickness change in children who liked active playing outside compared with other groups between the left and right arms over one year are unknown. However, considering the ulna muscle thickness includes two major extrinsic flexor muscles of the fingers, whereas the radius muscle thickness involves muscles in the forearm pronation and wrist and elbow joint flexion [47], and differences in how to use the left and right hands may have appeared (e.g., perhaps related to tasks completed with dominant vs. non-dominant hands; most children were right-handed). These are all speculations and future research is needed to clarify this issue.

### 4.3. Physical Activity Assessment

In this study, class teachers assessed their children’s physical activity during their free time in a kindergarten, as reported previously [48]. The teachers spend time in kindergarten with the children in their class (approximately between 8:00 AM and 3:00 PM) every weekday. An advantage of the proxy-reported tools over the other measurement tools, such as the accelerometer, is that they could capture the context and type of the behaviors [49]. This study aimed not to quantify children’s physical activity (such as intensity and duration) but to understand the kind of play that children prefer. From this point, it is considered that the assessments of physical activity by the teachers were appropriate for measuring the behaviors of interest. Although we view this assessment as appropriate and informative, subjective assessments of physical activity from teachers are imperfect.

## 5. Conclusions

Free play in kindergarten is roughly divided into fine and gross motor activities. This study observed no difference in one-year changes in handgrip strength between activity groups, but children who liked active playing outside were stronger than other children. These results suggest that there are already differences in strength in young children prior to entering kindergarten. Assuming these children had similar activity levels prior to entering school, these results suggest that children who actively play outdoors may acquire higher handgrip strength than those who prefer indoor play. Regardless of the reason, strength differences between children appear very early in life. Furthermore, changes in forearm muscle thickness might be inconsistent with handgrip strength changes among the three activity groups.

## Figures and Tables

**Table 1:** Changes in anthropometric variables, ultrasound-measured forearm muscle thickness, and handgrip strength in boys and girls.

	Boys	Girls
N	49	46
Age (yr)		
Test 1	4.5 ± 0.6	4.6 ± 0.5
Test 2	5.5 ± 0.6	5.6 ± 0.5
Height (cm)		
Test 1	103.9 ± 5.5	103.1 ± 5.0
Test 2	109.9 ± 5.6	109.3 ± 5.4
Change	6.0 ± 0.8	6.2 ± 0.9
Body mass (kg)		
Test 1	17.0 ± 1.9	16.4 ± 2.1
Test 2	18.8 ± 2.4	18.3 ± 2.7
Change	1.8 ± 0.9	1.9 ± 0.9
Muscle thickness ulna, right hand (mm)		
Test 1	21.2 ± 1.7	20.8 ± 2.0
Test 2	23.3 ± 1.7	22.6 ± 2.1
Change	2.0 ± 0.9	1.8 ± 1.3
Muscle thickness radius, right hand (mm)		
Test 1	10.6 ± 1.3	10.1 ± 1.3
Test 2	11.3 ± 1.3	10.8 ± 1.5
Change	0.7 ± 0.8	0.7 ± 0.9
Handgrip strength, right hand (kg)		
Test 1	8.1 ± 2.7	6.6 ± 2.4
Test 2	11.6 ± 2.3	9.8 ± 2.3
Change	3.5 ± 1.6	3.3 ± 2.1

Results are expressed as mean and standard deviation. Statistical differences are noted in the text. The table is meant to be descriptive.

**Table 2:** Changes in anthropometric variables, forearm muscle thickness, and handgrip strength in three groups.

	Group 1	Group 2	Group 3
N	23 (9 boys, 14 girls)	34 (15 boys, 19 girls)	38 (25 boys, 13 girls)
Age (yr)			
Test 1	4.4 ± 0.6	4.6 ± 0.5	4.7 ± 0.6
Test 2	5.4 ± 0.6	5.6 ± 0.5	5.7 ± 0.5
Height (cm)			
Test 1	101.9 ± 4.8	103.1 ±5.0	104.9 ± 5.5
Test 2	108.1 ± 5.4	109.1 ± 5.2	110.9 ± 5.5
Body mass (kg)			
Test 1	16.6 ± 2.4	16.1 ± 1.7	17.3 ± 2.0
Test 2	18.4 ± 3.0	17.9 ± 2.2	19.3 ± 2.5
Muscle thickness ulna, right hand (mm)			
Test 1	20.7 ± 2.2	20.7 ± 1.8	21.4 ± 1.6
Test 2	22.4 ± 2.3	22.4 ± 1.7	23.7 ± 1.6
Change	1.7 ± 1.2	1.7 ± 1.1	2.3 ± 1.0
Muscle thickness ulna, left hand (mm)			
Test 1	20.7 ± 2.2	20.3 ± 1.5	21.2 ± 1.7
Test 2	22.4 ± 2.4	22.1 ± 1.8	23.3 ± 1.7
Change	1.7 ± 1.1	1.8 ± 1.0	2.1 ± 1.4
Muscle thickness radius, right hand (mm)			
Test 1	10.0 ± 1.4	10.1 ± 1.4	10.7 ± 1.0
Test 2	10.7 ± 1.8	10.6 ± 1.4	11.6 ± 1.0
Change	0.7 ± 1.0	0.5 ± 0.7	0.9 ± 0.9
Muscle thickness radius, left hand (mm)			
Test 1	10.4 ± 1.5	9.7 ± 1.5	10.8 ± 1.1
Test 2	10.7 ± 1.6	10.6 ± 1.4	11.7 ± 1.2
Change	0.3 ± 1.0	0.9 ± 0.9	0.9 ± 0.9
Handgrip strength, right hand (kg)			
Test 1	6.1 ± 2.2	7.2 ± 2.8	8.2 ± 2.5
Test 2	9.7 ± 2.6	10.2 ± 2.5	11.8 ± 1.9
Change	3.6 ± 2.0	3.0 ± 1.7	3.6 ± 2.0
Handgrip strength, left hand (kg)			
Test 1	5.7 ± 2.4	6.8 ± 2.6	7.5 ± 2.5
Test 2	8.9 ± 2.8	9.5 ± 2.3	10.8 ± 1.8
Change	3.2 ± 2.0	2.7 ± 1.5	3.4 ± 1.8

Results are expressed as mean and standard deviation. Change = Test 2 − Test 1. Statistical differences are noted in the text. The table is meant to be descriptive.

## Data Availability

Data are available from the corresponding author upon reasonable request.
